# Preterm Labor and Maternal Hypoxia in Patients With Community-Acquired Pneumonia

**DOI:** 10.1155/S1064744996000427

**Published:** 1996

**Authors:** Maurizio L. Maccato, Phillip Pinell, Mark G. Martens, Sebastian Faro

**Affiliations:** 1 Department of Pediatrics Baylor College of Medicine Houston TX USA; 2 Department of Microbiology and Immunology Baylor College of Medicine Houston TX USA; 3 Department of Obstetrics and Gynecology Hennepin County Medical Center Minneapolis MN USA; 4 Department of Gynecology and Obstetrics Rush University Rush Medical College Chicago IL USA; 5 Houston Perinatal Associates 7400 Fannin, #700 Houston TX 77054 USA

## Abstract

*Objective:* We sought to determine if preterm labor is associated with the degree of maternal hypoxia in pregnant women with community-acquired pneumonia but no other maternal diseases.

*Methods:* We retrospectively reviewed the medical records of all antepartum patients admitted with a diagnosis of community-acquired pneumonia to an inner-city university hospital between 1983 and 1987. Included in this review were only the patients with radiologically confirmed diagnose of pneumonia and documented arterial blood gases on room air at the time of admission, but no other maternal diseases.

*Results:* A total of 22 cases were identified. There was no maternal mortality, but there were 2 patients (9%) who developed respiratory failure requiring mechanical ventilation. Bacteremia with *Streptococcus pneumoniae* was documented in 1 patient (5%). Preterm labor complicated 5 cases (23%) and led to preterm delivery in 3 patients (14%). Terbutaline tocolysis was instituted in 3 patients, but was discontinued in 1 patient who was allowed to deliver because of her worsening condition. Preterm labor was associated with the WBC count on admission, usually > 18,000/mm^3^, but no statistically significant correlation with the severity of maternal hypoxia was noted. Five patients (23%) were incorrectly diagnosed at the time of admission, 4 with an initial diagnosis of pyelonephritis and 1 with an initial diagnosis of cholecystitis.

*Conclusions:* Community-acquired pneumonia in the antepartum period is responsible for significant maternal and fetal complications even in the absence of other maternal diseases. Preterm labor and delivery remain frequent, and tocolysis should be used cautiously. At the time of admission, the diagnosis may be difficult. The degree of maternal hypoxia on admission does not correlate with the presence of preterm labor.


Infectious Diseases in Obstetrics and Gynecology 4:221–224 (1996)
© 1997 Wiley-Liss, Inc.

### Preterm Labor and Maternal Hypoxia in Patients With Community-Acquired Pneumonia

Maurizio L. Maccato, Phillip Pinell, Mark G. Martens, and Sebastian Faro

*Departments of Pediatrics (M.L.M., P.P.) and Microbiology and Immunology (M.L.M.), Baylor College of Medicine, Houston, TX; Department of Obstetrics and Gynecology, Hennepin County Medical Center, Minneapolis, MN (M.G.M.); Department of Gynecology and Obstetrics, Rush University, Rush Medical College, Chicago, IL*

### Abstract

***Objective:*** **We sought to determine if preterm labor is associated with the degree of maternal hypoxia in pregnant women with community-acquired pneumonia but no other maternal diseases.**

***Methods:*** **We retrospectively reviewed the medical records of all antepartum patients admitted with a diagnosis of community-acquired pneumonia to an inner-city university hospital between 1983 and 1987. Included in this review were only the patients with radiologically confirmed diagnose of pneumonia and documented arterial blood gases on room air at the time of admission, but no other maternal diseases.**

***Results:*** **A total of 22 cases were identified. There was no maternal mortality, but there were 2 patients (9%) who developed respiratory failure requiring mechanical ventilation. Bacteremia with *Streptococcus pneumoniae* was documented in 1 patient (5%). Preterm labor complicated 5 cases (23%) and led to preterm delivery in 3 patients (14%). Terbutaline tocolysis was instituted in 3 patients, but was discontinued in 1 patient who was allowed to deliver because of her worsening condition. Preterm labor was associated with the WBC count on admission, usually >18,000/mm$^3$, but no statistically significant correlation with the severity of maternal hypoxia was noted. Five patients (23%) were incorrectly diagnosed at the time of admission, 4 with an initial diagnosis of pyelonephritis and 1 with an initial diagnosis of cholecystitis.**

***Conclusions:*** **Community-acquired pneumonia in the antepartum period is responsible for significant maternal and fetal complications even in the absence of other maternal diseases. Preterm labor and delivery remain frequent, and tocolysis should be used cautiously. At the time of admission, the diagnosis may be difficult. The degree of maternal hypoxia on admission does not correlate with the presence of preterm labor.** © 1997 Wiley-Liss, Inc.

Key words

Pregnancy, infection, complications, prematurity

Pneumonia in the antepartum period is uncommon. Prior reports have placed its frequency between 0.4% and 1%.[1] While the maternal mortality rate prior to the advent of effective antimicrobial therapy was reported as high as 24%,[2] the current mortality rates are much lower, frequently involving patients with other serious concomitant diseases.[3] However, preterm labor and preterm delivery are reported as frequent complications, again more commonly in patients with other medical problems. This retrospective review was performed to identify a possible correlation between the degree of mater-

Address correspondence/reprint requests to Dr. Maurizio L. Maccato, Houston Perinatal Associates, 7400 Fannin, #700, Houston, TX 77054.

Received September 5, 1995
Accepted September 16, 1996

Clinical Study nal hypoxia and the presence of preterm labor. Because preterm labor may be associated with other concomitant maternal illnesses, we limited our analysis to patients with community-acquired pneumonia who did not suffer from other acute or chronic conditions.

### MATERIALS AND METHODS

We retrospectively reviewed the medical records of all patients admitted between January 1983 and December 1987 to a university-affiliated community hospital (Jefferson Davis Hospital, Houston, TX) with a diagnosis of community-acquired pneumonia in the antepartum period. These patients were identified by a computer search of all admissions during that interval. We included in our review only those patients with dyspnea, cough, or pleuritic chest pain and the diagnosis of pneumonia confirmed by chest radiograph, in the absence of other concomitant medical conditions. The patients diagnosed with preterm labor had either documented cervical changes or at least 3 contractions in 10 min for 1 h. Terbutaline was administered as a subcutaneous injection of 0.25 mg every 15 min for 3 doses, followed by 5 mg orally every 4 h. All patients had gestational ages >20 weeks. Likewise, the patients who did not have arterial blood gases obtained at the time of diagnosis were excluded. No further attempt at identifying cases other than those on the computer-generated list was made.

The statistical analysis was performed using the 2-tailed Student's t-test, with $P < 0.05$ considered significant.

### RESULTS

A total of 22 patients fitting our inclusion criteria were identified. The mean age was 23.5 years (range 15–38 years) and the mean gestational age at the time of diagnosis was 29.4 weeks (range 22–38 weeks). Eight women (36%) were primigravidas, and 5 women (23%) were smokers. One woman had a history of alcohol abuse, and 1 woman had a twin gestation.

While, in retrospect, all of our patients had clinical presentations consistent with pneumonia, 4 patients (18%) had an admitting diagnosis of pyelonephritis or urinary-tract infection and 1 patient (5%) of cholecystitis. In these 5 women, the correct diagnosis was made 24–48 h after admission. On the chest radiographs, infiltrates were unilateral in 14 patients (64%) and bilateral in the remaining 8 patients (36%). The arterial blood gases on room air demonstrated an average pH of 7.43 (range 7.36–7.51), $pO_2$ of 70.5 mmHg (range 39–99 mmHg), and $pCO_2$ of 29.4 mmHg (range 20–37 mmHg). Five of our patients (23%) suffered from preterm labor noted on or shortly after admission. Three of them (14%) delivered prematurely, 2 at 28 weeks estimated gestational age and 1 at 34 weeks. The 2 other patients were successfully treated with terbutaline tocolysis at 28 and 31 weeks gestation. Terbutaline tocolysis was attempted in 1 of the patients who delivered at 28 weeks, but it was stopped after about 16 h because of the rapid deterioration of her condition requiring intubation and mechanical ventilation. This patient was allowed to deliver, 12 h after which her respiratory status significantly improved. Extubation was accomplished 36 h later. In this patient, the blood cultures were negative and sputum cultures revealed mixed flora. The other patient who required intubation and mechanical ventilation presented at 26 weeks gestation with a pH of 7.46, $pO_2$ of 39, and $pCO_2$ of 39. Again, a deterioration of the maternal condition required intubation and mechanical ventilation, but preterm labor was not noted and resolution of her condition did not require delivery of the infant. In this patient as well, the blood cultures were negative and sputum cultures did not identify a pathogen.

Table 1 lists the clinical parameters of patients with and without preterm labor. Only the WBC count on admission demonstrated a statistically significant difference. Among the patients who suffered from preterm labor, 2 had $pO_2$s of <60 mmHg on admission (40%), and only 1 (20%) had a WBC of <18,500/mm$^3$. Among the patients who did not suffer from preterm labor, 2 (12%) had $pO_2$s of <60 mmHg and 14 (82%) had WBC counts of <18,500/mm$^3$.

All patients had blood cultures drawn on admission. Five patients did not have sputum cultures collected prior to the institution of antimicrobial therapy. Three patients had sputum cultures that were judged not reliable because the sputum smear showed more than 5 epithelial cells per low power field. Among the 14 patients with sputum smears evaluable for microbiologic data, *Streptococcus pneumoniae* was isolated in 4 cases (29%) and *Haemophilus influenzae* and *Klebsiella pneumoniae* were recov-

TABLE 1. Clinical parameters of patients with and without preterm labor[a]

| | No preterm labor (N = 17) | | Preterm labor (N = 5) | | |
|---|---|---|---|---|---|
| | Mean | Standard deviation | Mean | Standard deviation | *P* (<0.05) |
| Age (years) | 24.2 | Range 16–38 | 21.4 | Range 15–29 | NS |
| EGA (weeks) | 29.3 | 6.0 | 29.8 | 2.4 | NS |
| Temperature on admission | 100.9°F (38.3°C) | 1.8 | 101.1°F (38.4°C) | 0.8 | NS |
| Hematocrit | 32.8 | 3.2 | 33.6 | 6.2 | NS |
| Hemoglobin (gm/dl) | 10.8 | 1.2 | 11.2 | 1.9 | NS |
| WBC count (1,000/mm$^3$) | 13.8 | 4.5 | 22.3 | 6.0 | 0.004 |
| Arterial blood gases (room air) | | | | | |
| ph | 7.43 | 0.03 | 7.44 | 0.04 | NS |
| $pO_2$ (mmHg) | 72.8 | 15.7 | 62.6 | 10.3 | NS |
| $pCO_2$ (mmHg) | 29.6 | 4.2 | 28.8 | 2.0 | NS |

[a]EGA = estimated gestational age. NS = not significant.

ered in 1 case each (7%). Cultures for mycoplasma, chlamydia and viruses, and serologies were not routinely performed. In 1 patient, the blood cultures were positive for *S. pneumoniae*, but the sputum cultures revealed only mixed flora. This patient did not experience preterm labor.

The antibiotic regimens used ranged from single-agent therapy with erythromycin, cefazolin, penicillin G, or ampicillin, to multiple-agent regimen, usually combining erythromycin with a penicillin or cephalosporin.

### DISCUSSION

In the population studied, even in the absence of complicating preexisting maternal diseases, pneumonia was associated with significant morbidity for both the mother and the fetus. The rate of preterm delivery during the episode of pneumonia in our patients (3/22; 14%) was similar to the rate reported by Madinger et al.[3] (4/19; 21%) in patients with no underlying maternal disease. However, the incidence of preterm labor was not as high in our series (5/22; 33%) as in the series of Madinger et al.[3] (9/19; 47%), perhaps because of different definitions of preterm labor.

The results of the sputum cultures were similar to prior reports, with *S. pneumoniae* being the most frequently isolated organism and *H. influenzae* and *K. pneumoniae* being less common isolates.[4,5]

Our hypothesis that hypoxia was associated with preterm labor or delivery could not be confirmed in our study. While there was a difference in the mean arterial oxygen pressure between the group of patients who suffered from preterm labor and those who did not, such difference did not reach statistical significance, possibly because of the small number of patients in our study (power 0.33). We could, however, demonstrate a statistically significant increase in the mean WBC count on admission in the group of patients in preterm labor, perhaps as a result of a more intense inflammatory response. We speculate that increased WBCs may be associated with higher levels of circulating prostaglandins, leading to preterm labor. A better understanding of the pathophysiology of preterm labor in these patients may lead to more effective and safer tocolytic modalities, perhaps involving prostaglandin inhibitors.

In our view, the pregnant patient with community-acquired pneumonia is not a good candidate for outpatient therapy. We feel that aggressive supportive therapy, in conjunction with effective empiric antibiotic coverage, offers the patient the best chance for an uncomplicated recovery. In our experience, tocolysis can be considered if preterm labor ensues and the degree of prematurity of the fetus warrants the risks to the mother, but close monitoring of the clinical course of the disease is imperative.

### REFERENCES

1. Maccato ML: Pneumonia and pulmonary tuberculosis in pregnancy. In Faro S (ed): Obstetric and gynecologic infections. Obstet Gynecol Clin North Am 16(2):417–430, 1989.
2. Finland M, Dublin TD: Pneumococcic pneumonias complicating pregnancy and the puerperium. JAMA 112: 1027–1032, 1939.
3. Madinger NE, Greenspoon JS, Ellrodt AG: Pneumonia during pregnancy: Has modern technology improved maternal and fetal outcome? Am J Obstet Gynecol 161(3):657–662, 1989.
4. Benedetti TJ, Valle R, Ledger WJ: Antepartum pneumonia in pregnancy. Am J Obstet Gynecol 144(4):413–417, 1982.
5. Hopwood HG: Pneumonia in pregnancy. Obstet Gynecol 25(6):875–879, 1965.
